# 3D Printing and Electrospinning of Drug- and Graphene-Enhanced Polycaprolactone Scaffolds for Osteochondral Nasal Repair

**DOI:** 10.3390/ma18081826

**Published:** 2025-04-16

**Authors:** Izabella Rajzer, Anna Kurowska, Anna Nikodem, Jarosław Janusz, Adam Jabłoński, Magdalena Ziąbka, Elżbieta Menaszek, Jana Frankova, Wojciech Piekarczyk, Janusz Fabia

**Affiliations:** 1 Department of Mechanical Engineering Fundamentals, Faculty of Mechanical Engineering and Computer Science, University of Bielsko-Biala, 43-309 Bielsko-Biala, Poland; akurowska@ubb.edu.pl (A.K.); jjanusz@ubb.edu.pl (J.J.); ajablonski@ubb.edu.pl (A.J.); 2 Department of Mechanics, Materials and Biomedical Engineering, Faculty of Mechanical Engineering, Wroclaw University of Science and Technology, 50-372 Wroclaw, Poland; anna.nikodem@pwr.edu.pl; 3 Department of Ceramics and Refractories, Faculty of Materials Science and Ceramics, AGH University of Krakow, 30-059 Krakow, Poland; ziabka@agh.edu.pl; 4 Department of Cytobiology, Collegium Medicum, Jagiellonian University, 31-007 Krakow, Poland; elzbieta.menaszek@uj.edu.pl; 5 Department of Medical Chemistry and Biochemistry, Faculty of Medicine and Dentistry, Palacký University Olomouc, 775 15 Olomouc, Czech Republic; jana.frankova@upol.cz; 6 Department of Glass Technology and Amorphous Coatings, Faculty of Materials Science and Ceramics, AGH University of Krakow, 30-059 Krakow, Poland; wpiekar@agh.edu.pl; 7 Faculty of Materials, Civil and Environmental Engineering, University of Bielsko-Biala, 43-309 Bielsko-Biala, Poland; jfabia@ubb.edu.pl

**Keywords:** polycaprolactone, 3D printing, electrospinning, bi-layer scaffold, drug, graphene

## Abstract

A novel bi-layered scaffold, obtained via 3D printing and electrospinning, was designed to improve osteochondral region reconstruction. The upper electrospun membrane will act as a barrier against unwanted tissue infiltration, while the lower 3D-printed layer will provide a porous structure for tissue ingrowth. Graphene was integrated into the scaffold for its antibacterial properties, and the drug Osteogenon^®^ (OST) was added to promote bone tissue regeneration. The composite scaffolds were subjected to comprehensive physical, thermal, and mechanical evaluations. Additionally, their biological functionality was assessed by means of NHAC-kn cells. The 0.5% graphene addition to PCL significantly increased strain at break, enhancing the material ductility. GNP also acted as an effective nucleating agent, raising crystallization temperatures and supporting mineralization. The high surface area of graphene facilitated rapid apatite formation by attracting calcium and phosphate ions. This was confirmed by FTIR, µCT and SEM analyses, which highlighted the positive impact of graphene on mineral deposition. The synergistic interaction between graphene nanoplatelets and Osteogenon^®^ created a bioactive environment that enhanced cell adhesion and proliferation, and promoted superior apatite formation. These findings highlight the scaffold’s potential as a promising biomaterial for osteochondral repair and regenerative medicine.

## 1. Introduction

Nose injuries, including damage to the osteochondral region, present a growing clinical challenge with significant implications for both functional health and aesthetic outcomes [1]. These injuries, which can result from trauma, congenital defects, or degenerative processes, increasingly affect individuals across all age groups. The osteochondral region in the nose varies in thickness and material composition. This complexity makes it challenging to accurately repair and restore nasal function during surgical procedures [2]. Nasal injuries can compromise functional integrity of the nasal airway, leading to a range of respiratory issues. Disruption of the osteochondral region can result in nasal obstruction, impaired airflow, and chronic sinusitis. Injuries to the nasal septum, which include both bone components (e.g., the perpendicular plate of the ethmoid bone and the vomer) and cartilage elements (e.g., the septal cartilage), can lead to a deviated septum [3]. Such a deviation obstructs nasal passages and exacerbates respiratory difficulties. Repairing osteochondral defects of both cartilage and bone often requires employing biomaterials to support tissue regeneration and restore function [4]. Ideal scaffolds should be biocompatible and biodegradable. They should possess strong and stable mechanical properties and exhibit good adhesion to bone-forming cells, which must migrate to the scaffold, attach and proliferate there. Additionally, a key feature of the scaffold are interconnected pores, which enable cells to grow and distribute evenly throughout the porous structure, facilitating angiogenesis and integration with the surrounding tissue [5]. Although current implant materials for the reconstruction of nasal defects have shown promising results, in recent years the number of reported implant failures or malfunctions due to bacterial infections have increased [6]. The respiratory system is often exposed to bacteria and viruses and the consequences of infections related to the implant’s presence in the organism are significant. They usually require revision surgery, implant removal, prolonged antibiotic treatment and, in extreme cases, can even lead to death. Therefore, it is highly beneficial to endow biomaterials used for nasal implants with antibacterial properties. Numerous techniques are employed to produce 3D scaffolds, including both traditional methods [7,8,9,10] and those based on additive manufacturing technologies [11,12]. A properly selected technology plays a vital role in tissue engineering and regenerative medicine. This provides possibilities to create structures that mimic the natural extracellular matrix, offering a suitable environment for cell growth and differentiation [13]. Recent developments in additive manufacturing technologies allow for the creation of implants or scaffolds customized to the specific anatomical and functional needs of individual patients. These advancements also provide precise control over the implants’ external shape and internal porosity [14,15].

Fused Deposition Modeling (FDM) is a popular 3D printing technique used to fabricate complex structures from thermoplastic materials [16]. In FDM, a filament of material is melted and extruded through a nozzle to build up a structure layer by layer. This method is advantageous due to its precision, scalability and cost-effectiveness.

Electrospinning (ES) is a technique used to produce microfibers and nanofibers from a wide variety of materials, including polymers, ceramics and composites. For instance, fibrous membranes that closely resemble the native extracellular matrix (ECM) are manufactured via ES [17,18]. Electrospun fibrous membranes offer distinct advantages such as: a large surface area-to-volume ratio, high porosity, uniform fiber size, diverse composition, structural flexibility and ease of functionalization with bioactive molecules [19]. These features make them promising candidates for drug delivery applications [20]. Membranes acting as barriers to bacterial flora can aid in the infection-free healing of defects [21,22,23]. Additionally, these membranes can separate parts of an implant, preventing the ingrowth of unwanted surrounding tissue. They can also enhance bioactivity and promote osseointegration with surrounding tissues via bioactive powders incorporated into an electrospinning solution.

Combining FDM with electrospinning may potentially further enhance scaffold properties.

Polycaprolactone (PCL) is a biodegradable polymer with excellent processing properties. Using FDM 3D printing technique, customized PCL scaffolds with specific shapes, porosity, or pore sizes are obtained, while maintaining proper mechanical strength [24]. However, PCL’s highly hydrophobic nature hinders cell adhesion and proliferation on its surface [25]. Additionally, PCL and its degradation products do not inherently promote significant osteogenesis when used alone. Some studies suggest that incorporating into PCL other substances, such as bioglass, hydroxyapatite, or Osteogenon^®^ (OST, PIERRE FABRE, Warsaw, Poland), can enhance its wettability, degradability and biocompatibility [26,27].

Graphene and its derivatives have attracted significant attention due to their unique physical, chemical, electrical and mechanical properties [28]. They constitute a highly promising class of antimicrobial materials due to diverse bactericidal mechanisms and relatively low cytotoxicity [29,30]. Several mechanisms have been proposed to explain the antimicrobial effectiveness of graphene materials. One such mechanism involves the physical damage of bacterial membranes with graphene sharp edges, leading to cell death [31]. Graphene can also inhibit bacterial proliferation by inducing oxidative stress and the subsequent microbial DNA damage. Other mechanisms include wrapping and photothermal ablation [32]. Due to graphene superior antibacterial properties, good biocompatibility and adequate mechanical strength, graphene-based composites are suitable for producing nasal implants for the reconstruction of osteochondral defects.

Osteogenon^®^ (OST) is a pharmacological compound commonly used in the treatment of bone disorders. Osteogenon^®^ comprises ossein, a protein-rich component derived from bone matrix, and hydroxyapatite [33]. The ossein component contains collagen and other non-collagenous proteins vital for bone metabolism. OST is often prescribed to manage osteoporosis and to facilitate the healing of fractures, as it provides both organic and inorganic elements crucial for bone formation and maintenance [34]. OST’s efficiency consists of the synergistic effect of ossein and hydroxyapatite, offering a balanced source of calcium, phosphate, collagen, and growth factors. This combination stimulates osteoblasts and inhibits the activity of osteoclasts, thereby supporting bone regeneration and strengthening [35]. OST has good biocompatibility and efficient absorption in the body, making it an effective means of delivering essential nutrients and components directly to bone tissue.

Despite ongoing advances in biomaterials and scaffold fabrication techniques, several critical challenges persist in the field of osteochondral tissue engineering. These include achieving strong integration at the cartilage–bone interface, ensuring mechanical stability under physiological loads, preventing bacterial infections, and providing a microenvironment conducive to both chondrogenic and osteogenic differentiation. Recent studies have emphasized the importance of developing composite scaffolds that combine antibacterial function with bioactivity to promote multi-tissue regeneration [36,37,38]. However, many current approaches either fail to sufficiently mimic the complex osteochondral interface or lack adaptability to patient-specific needs.

To address these limitations, our study proposes a novel, dual-functional scaffold design that integrates 3D printing and electrospinning technologies, enhanced with graphene nanoplatelets (GNP) for antibacterial protection and Osteogenon^®^ (OST) for osteoinductive support. This layered approach aims to replicate the anatomical and functional complexity of the nasal osteochondral region while improving tissue integration and regeneration. The upper layer consists of an electrospun membrane that acts as a barrier to prevent the infiltration of surrounding tissues, while the lower layer, produced via 3D printing, provides optimal porosity to facilitate tissue growth. Additionally, the scaffold is modified with graphene to confer antibacterial properties, and Osteogenon^®^ is incorporated to accelerate bone tissue regeneration. The fabricated scaffolds were characterized physically, thermally, and mechanically. Finally, NHAC-kn cells were used to evaluate the functionality of the layered composite scaffolds. By leveraging the customizable nature of additive manufacturing, the proposed scaffold holds potential for future personalization of implants tailored to individual patients.

## 2. Materials and Methods

### 2.1. Materials

Polycaprolactone pellets (80 kDa) were purchased from Sigma Aldrich, Gillingham, UK. The PCL filaments were produced as sticks using an injection molding Babyplast machine Rambaldi+CO I.T. Srl, Molteno, Italy, as described in our earlier work [39]. The filaments were short sticks with a diameter of 1.75 mm. Their ends were to connect to each other, thus enabling the construction of scaffolds made from various materials. In this study, we focused on fabricating a graphene-modified filament suitable for 3D printing to produce a material for nasal implants. In order to enhance antibacterial properties we used graphene nanoplatelets (GNP, polycarboxylate functionalized, hydrophilic, Sigma Aldrich, UK). Based on the results of our previous research [39], 0.5 wt%. GNP was added during the production process of the filament sticks. PCL combined with a small concentration of GNP demonstrated notable antibacterial effectiveness, particularly against *C. albicans* and *S. aureus*. However, with the graphene content increase (5%, 10 wt%), the samples’ antibacterial effectiveness deteriorated and the microorganism proliferation even rose [40]. A pure PCL filament was used as a reference.

Scaffolds were fabricated using the FDM technique with a commercial 3D printer (Anet A8, Anet Technology Co., Ltd., Shenzhen, China). The scaffold model consisted of three layers of parallel bars with a square cross-section of 1 mm side length, spaced 0.7 mm apart. The bars in adjacent layers were oriented perpendicularly, creating cube-shaped pores. The printing parameters were as follows: the nozzle temperature 190 °C, the print bed temperature 50 °C, and the layer thickness set to 0.2 mm. Next, the PCL and PCL_GNP samples were positioned on the collector of the electrospinning machine.

Osteogenon^®^ drug (OST, PIERRE FABRE, Warsaw, Poland) was used as an additive in electrospinning. Each tablet contained 830 mg of the active substance, which included approx. 444 mg hydroxyapatite, 178 mg calcium, and 82 mg phosphorus. PCL (8 wt%) was dissolved in a 1:1 volume ratio mixture of methanol and chloroform. OST particles (1.5 wt%) were dispersed in the solution using an ultrasonic shaker. The solution was then stirred with a magnetic stirrer for 24 h to dissolve PCL and form a stable suspension. The production of nanofibrous membranes via electrospinning is influenced by various operating parameters, including the intrinsic properties of the solution, operational conditions, and ambient parameters. In our work, optimal parameters for forming uniform fibers were determined by adjusting the process settings. The preferred voltage, flow rate, and tip-to-collector (in this case, a 3D scaffold) distance were: 20 kV, 1.5 mL/h, and 20 cm, respectively. A G25 gauge needle was used, and the process was conducted using a laboratory electrospinning setup (TIC 1092012 machine, UBB, Bielsko-Biała, Poland). The scaffolds had a top layer of PCL_OST nanofibers and a bottom layer of 3D-printed porous structures made from PCL and PCL_GNP. Two types of the layered materials were produced: (1) PCL/PCL_OST and (2) PCL_GNP/PCL_OST.

### 2.2. Analysis and Testing

The scaffold’s macrostructure and microstructure were assessed using a stereomicroscope and an optical microscope (Opta-Tech, Warsaw, Poland) fitted with a CMOS 3 camera and OptaView IS v7 software. The scanning electron microscope (Apreo 2 S LoVac, Thermo Fisher Scientific, Waltham, MA, USA) was used to observe morphology. The polymeric and composite samples before and after incubation in simulated body fluid (SBF) were placed on a conductive carbon tape and coated with a 10 nm carbon layer (EM ACE600 sputter coater, Leica Microsystems, Wetzlar, Germany) and then observed at an accelerated voltage of 10–18 kV, under low vacuum, using the secondary electron mode.

FTIR spectra were recorded over the wavenumber range of 400–4000 cm^−1^, with 32 scans at a resolution of 4 cm^−1^, using a Thermo Scientific Nicolet iS5 FT-IR Spectrometer, (Thermo Fisher Scientific Inc., Waltham, MA, USA) equipped with an iD7 ATR module featuring a diamond crystal for Attenuated Total Reflectance (ATR).

Calorimetric (DSC) investigations were carried out with an analytical system (TA Instruments, New Castle, DE, USA) equipped with a MDSC Calorimeter 2920 and a refrigerated cooling system. The samples were heated at the rate of 10 °C/min from −5 °C to approximately 250 °C, and then cooled at the same rate. The other conditions of measurements were as follows: atmosphere N2, purge 40 cm^3^/min; standard aluminum pans. The examined samples weight amounted to approximately 5 mg. Registration sensitivity was above 0.2 μW. The DSC curves analysis, involving the determination of enthalpies and characteristic temperatures of transitions, was performed with Universal V4.5A software supplied by TA Instruments.

The registrations of each scaffold and the structural parameters measurements were conducted using non-invasive computed microtomography (1172 SkySkan, Bruker, Kontich, Belgium). Each scaffold was recorded at a resolution of 6 µm, with X-Ray tube parameters of 38 kV and 240 µA, 360° registration with a rotation angle of 0.4° and exposure time of 345 ms.

The samples were reconstructed using the NRecon 2.0 software (Bruker, Kontich, Belgium), after which qualitative and quantitative studies were conducted using the CTVox and CTAn software (Bruker, Kontich, Belgium), respectively. Importantly, the membrane and the scaffold bottom layer were measured separately. The structural parameters were obtained via the 3D reconstruction generated with image analysis algorithms (segmentation, thresholding; Otsu method) and dedicated software (CtAn, Bruker). Due to such measurements it was possible to determine: structure porosity (Po.tot), individual phases proportion, and structure thickness (St.Th).

The scaffolds’ mechanical properties, such as tensile strength, Young’s modulus and strain at break, were evaluated using a Universal Testing Machine (Hegewald & Peschke, Nossen, Germany) with a crosshead speed of 5 mm/min. The specimen dimensions were 3 × 7 × 20 mm (thickness × width × length). For each scaffold type, 6 independent measurements were performed.

The scaffolds were incubated at 37 °C for 14 days in a 1.5× SBF solution prepared according to the previously described method [41]. On day 1, 7, and 14, the scaffolds were removed, rinsed with DI water, and examined using SEM, EDS and µCT.

### 2.3. Cell Culture

Cell cultivation—Normal Human Knee Articular Chondrocytes (NHAC-kn, Lonza, Switzerland) were cultivated in a cell culture medium (Chondrocyte Growth Medium CGMTM, Lonza, Switzerland) supplemented with 5% fetal bovine serum (FBS) and growth factors (CGMTN SingleQuotsTM Kit, Lonza, Switzerland) at humidified atmosphere (5% CO_2_ and 37 °C) based on the manufactured protocol. The culture medium was changed every 3 days to reach 85% confluence, and the cells were used for subculture. Briefly, the medium was aspirated from cultivation flask and trypsin/EDTA solution was added till the cells were detached. The trypsinization was allowed till 90% of cells were rounded. After cell release, the neutralization solution was added and dilute the cells were used for further cultivation. The 2nd passage of cells was used for all experiments.

Scaffold sterilization—the samples (pure PCL, PCL/PCL_OST and PCL_GNP/PCL_OST) were incubated in 70% ethanol under UV irradiation for 1.5 h on each side. The materials were placed at the well bottom (Nunc, Roskilde, Denmark), cells seeded at final density 2 × 10^4^ cells/well and cultivated for 7, 14 and 28 days in a differentiation medium (Chondrocyte Differentiation Medium CDMTM, Lonza, Switzerland) with supplement (CDMTM BulletKitTM, Lonza, Switzerland), TGF-β3, ascorbic acid.

Cell viability was determined by Presto BlueTM Cell Viability Reagent (Thermo Fisher Scientific, USA), resazurin-based reagent, after 7 and 28 days, according to the manufacturer protocol. Since no lysis is required, the diluted Presto BlueTM solution can be removed from the cells and replaced with complete growth medium for further culturing and use in downstream assays. These nontoxic and cell-permeable reagents quantitatively measure cell viability in as little as 10 min using fluorescence-based microplate readers. The fluorescence was measured on an Omega microplate reader (BMG Labtech, Ortenberg, Germany).

Measurement of adenylate cyclase level—the luminometric determination of the adenylate cyclase level that corresponded to the cells number (proliferation) was conducted via ToxiLight^TM^ Non-destructive Cytotoxicity BioAssay Kit (Lonza, Basel Stucki, Switzerland), according to the manufacturer protocol. The 0.001% resazurin was added to each well at inoculation, then, after incubation, fluorescence was measured with excitation at 570 nm and emission at 615 nm. Each assay was performed in triplicate.

Statistical Analysis—data for statistical analysis were presented as the mean ± SD of at least 3 independent experiments. Significance was determined with unpaired *t*-test. *p*-values of 0.05 or less were considered as statistically significant.

## 3. Results

**Figure 1:**
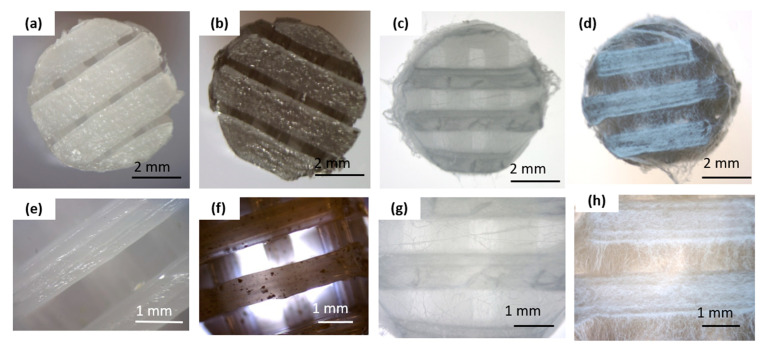
Macro- and microscopic characterization of the scaffold before and after electrospinning: (**a**,**e**) pure PCL 3D-printed scaffold; (**b**,**f**) graphene-modified 3D-printed scaffold (PCL_GNP); (**c**,**g**) 3D-printed PCL scaffold with electrospun PCL_OST membrane (PCL/PCL_OST); (**d**,**h**) 3D-printed PCL_GNP scaffold with electrospun PCL_OST membrane (PCL_GNP/PCL_OST). Stereoscopic microscope images (**a**–**d**) and optical microscope images (**e**–**h**).

Figure 1 summarizes the macro and microscopic evaluations conducted on the obtained scaffolds before and after the electrospinning process.

The bottom layer of the implants was produced using a 3D printing method (Figure 1a,b,e,f). The scaffolds displayed 3D porous microstructure. In the PCL_GNP samples (Figure 1f) graphene was noted, with the GNP particles well-dispersed throughout the scaffold and some small agglomerates. The fibrous membrane was successfully fabricated by electrospinning from the OST-modified PCL solution onto the surface of the printed scaffolds (Figure 1c,d,g,h).

**Figure 2:**
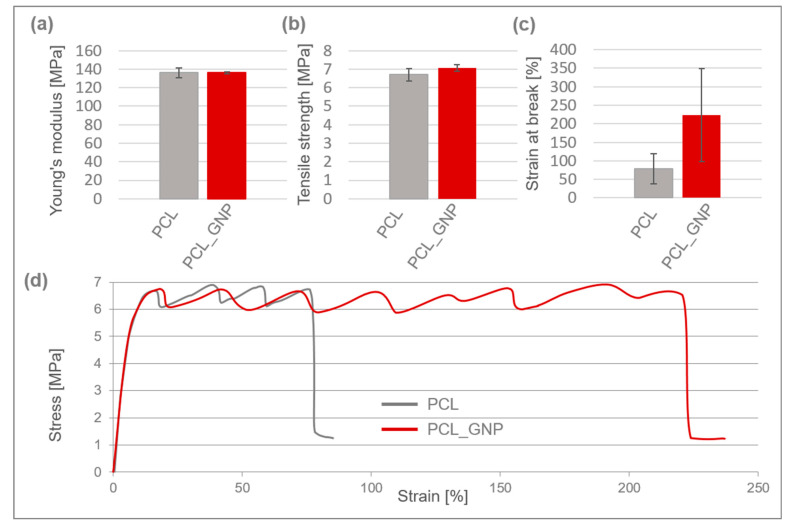
Mechanical properties of 3D-printed PCL and PCL_GNP scaffolds: (**a**) Young’s modulus; (**b**) tensile strength; (**c**) strain at break; (**d**) typical stress–strain curves.

Mechanical properties of 3D-printed PCL and PCL_GNP scaffolds are shown in Figure 2.

Both scaffolds exhibited typical plastic behavior, characterized by yielding, cold crystallization, and fracture, and they had a comparable Young’s modulus of approx. 136 MPa. The PCL_GNP sample demonstrated a slightly higher tensile strength of around 7.1 MPa.

After the printing process, the scaffolds’ tensile strength decreased by 59% for PCL and 57% for PCL_GNP, as compared to the filaments they were made from [39]. The reduction in tensile strength is likely due to thermal degradation and structural changes during the 3D printing process, which may weaken the molecular alignment compared to the original filaments. The strain at break for the PCL_GNP scaffold was approximately 3 times higher than that of the pristine PCL one. This suggests that the GNP addition resulted in higher tensile strength and elongation, and strain at break.

**Figure 3:**
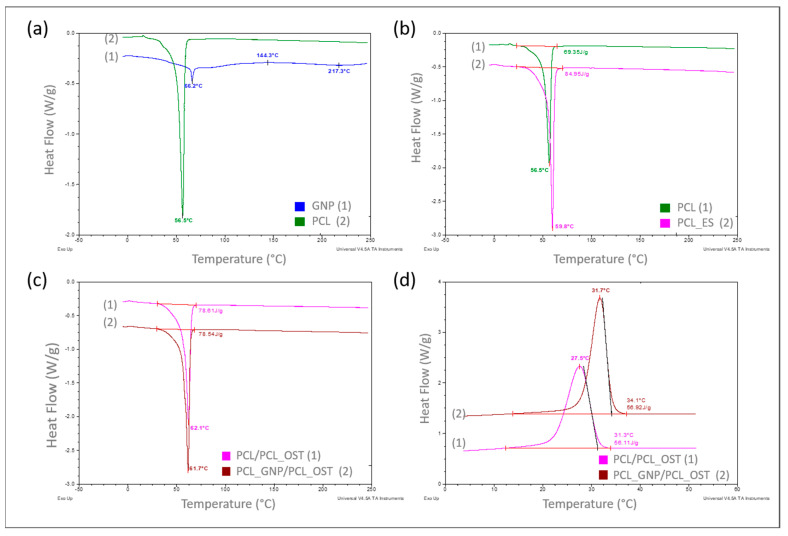
DSC curves recorded over a specified temperature range at a heating or cooling rate of 10°/min in a nitrogen atmosphere (flow 40 mL/min), respectively for samples: (**a**) PCL base polymer (2) and GNP used as a modifier (1)—heating mode; (**b**) PCL samples in granular (1) and ES membrane (2) form—heating mode; (**c**) with PCL_OST membrane applied to a scaffold printed from: PCL- (1) and GNP-modified PCL (2)—heating mode; (**d**) after melting 3D scaffolds printed from PCL (1) and PCL/GNP (2) with membrane PCL/OST layer applied—cooling mode.

The DSC results are presented concisely in Figure 3a–d, showing summaries of the DSC curves recorded as a function of the measured samples temperature.

The curves in Figure 3a–c refer to the measurements carried out in a heating mode, while the curves in Figure 3d were recorded in a controlled cooling mode. A comparison of the DSC curves is shown in Figure 3a. The curve recorded for the base polymer, polycaprolactone (PCL) in a granulate form (2), showed only one very distinct endothermic effect, corresponding to the melting of the crystalline phase, with a minimum at 56.5 °C. The curve recorded for graphene (GNP), used as a modifier (1), exhibited two endothermic effects with minima at 66.2 °C and 217.3 °C, respectively, and one exothermic effect at 144.3 °C, all of which are of low intensity. Such effects likely resulted from the thermal transitions of the functional groups used for graphene functionalization. With a small amount of the GNP additive (0.5%) used to modify the polycaprolactone, these effects did not appear at all on the DSC curve recorded for the modified PCL (Figure 3c).

In the next stage of this study, the impact of the form of polycaprolactone on its supermolecular structure was assessed. Figure 3b presents the DSC curve for PCL in a granulate form (discussed earlier) and the curve for PCL in the form of a membrane produced by electrospinning (2). It was found that the melting enthalpies for the two different forms of polycaprolactone differed significantly, at 69.35 J/g and 84.95 J/g, respectively.

Based on the following Equation (1), the crystallinity degree of polycaprolactone in both forms was determined:

(1) $$\kappa =\frac{\Delta H_{m}}{\Delta H^{0}}\cdot 100\%$$

where

ΔHm—the melting enthalpy of the sample (measured value), and

ΔH^0^—the melting enthalpy of pure 100% crystalline PCL, i.e.,139.4 J/g [42].

The degrees of calculated crystallinity were 49.7% for PCL and 60.9% for PCL_ES. The explanation for such a significant increase (over 11%) in the crystalline phase content in the PCL membrane can only be one—a strong influence of orientation occurring during the nanofibers formation, which later made up the membrane, in the electrostatic field during electrospinning.

The GNP influence on the crystalline structure of PCL was also examined during this study. The scaffolds with nanofibrous PCL membranes, and with the Osteogenon^®^ addition were studied (Figure 1c). The scaffolds differed in terms of the material they were printed from. Yet, the melting enthalpy for the pure PCL scaffold (1) was 78.61 J/g and the PCL scaffold modified with GNP (2) revealed almost the same value of 78.54 J/g. Using the equation (1), the crystalline phase content was calculated to be 56.3%. The crystallinity degree for both samples differed by less than 0.05%, which was significantly below the error margin. Only the 0.4 °C decrease in the minimum melting peak temperature occurred in the GNP sample, which correlated with the mean size of crystallites. This phenomenon was most likely caused by slightly faster cooling of the scaffold at the same printer nozzle temperature due to GNP better thermal conductivity. In the faster-cooling material, smaller crystallites formed and melted at a lower temperature when reheated in the DSC tests. The GNP additive had no influence on the PCL crystalline phase content, which was confirmed through calorimetric studies of non-isothermal crystallization from the discussed samples melt (Figure 1d). The specific enthalpy of transition for both samples was practically identical, with the difference of less than 1 J/g. It should be noted, however, that on the DSC curve for the GNP sample, the crystallization exothermic transition was clearly shifted towards higher temperatures. The temperature of the maximum peak was higher by 4.2 °C, and the onset transition temperature—corresponding to the nucleation temperature—was higher by 2.8 °C. This clearly proved GNP to be an effective heterogeneous nucleating agent in the crystallization process of PCL.

**Figure 4:**
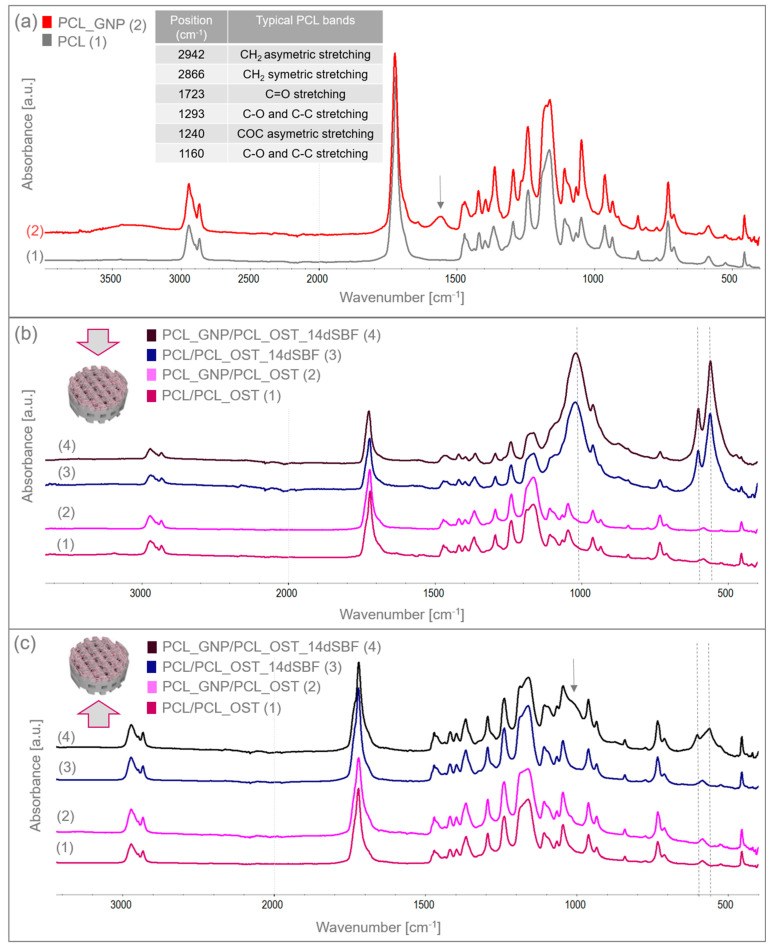
FTIR spectra of (**a**) 3D-printed PCL and PCL_GNP scaffolds; (**b**) layered samples before and after SBF immersion, analyzed from the membrane side; (**c**) layered samples before and after 14-day immersion in SBF, analyzed from the scaffold side.

The structure and functional groups of both the unmodified PCL and the GNP-modified scaffolds were analyzed using FTIR spectroscopy. Figure 4a shows the FTIR spectrum of the 3D-printed samples scanned from 400 to 4000 cm^−1^. The table in Figure 4a summarizes the characteristic bands of the unmodified PCL [33].

The GNP chemical composition included functional groups that overlapped with the PCL bands. However, the increased intensity of certain bands proved the GNP presence. For the PCL_GNP sample, new bands were observed: a broad band at 1555 cm^−1^, a small band at 1640 cm^−1^, and additional bands at 913 and 815 cm^−1^. Changes were also noted in the PCL bands at 1178, 1470, and 1266 cm^−1^, where band broadening was observed. The bands at 1555 cm^−1^ and 1640 cm^−1^ were attributed to stretching vibrations of C=C and aromatic groups associated with GNP [43,44]. The bands at 1266 cm^−1^ and 1178 cm^−1^ corresponded to C-O stretching vibrations and C-O-C stretching vibrations in PCL, respectively. The band at 1470 cm^−1^ corresponded to C=C skeletal vibrations in the graphitic domain [45]. The broad band observed at 3200–3500 cm^−1^ represented O-H stretching vibrations of hydroxyl groups, due to residual water molecules adsorbed on the surface.

The samples were immersed in simulated body fluid (SBF) and stored for 14 days to evaluate their bioactivity. Having been incubated, the samples were thoroughly rinsed with distilled water to remove residual fluid, then dried under controlled conditions, and subsequently subjected to the FTIR analysis. The FTIR results for the layered scaffolds from both sides of the samples are shown in Figure 4b,c. Figure 4b presents the analysis conducted from the membrane side, while Figure 4c displays the analysis from the printed scaffold side. In addition to the bands characteristic of PCL, the FTIR spectra also revealed bands corresponding to apatite at 559 and 600 cm^−1^, coming from deformation vibrations of PO_4_^3−^, and a wide band at 900–1200 cm^−1^ associated with stretching vibration of PO_4_^3−^. These bands were very small in the samples before SBF immersion. After 14 days of incubation, these bands increased significantly, indicating the apatite growth on the surface of the OST-modified membranes. Moreover, in the PCL_GNP/PCL_OST sample analyzed from the scaffold side (Figure 4c), these bands were also present, suggesting the graphene’s beneficial effect on the apatite growth during incubation.

**Figure 5:**
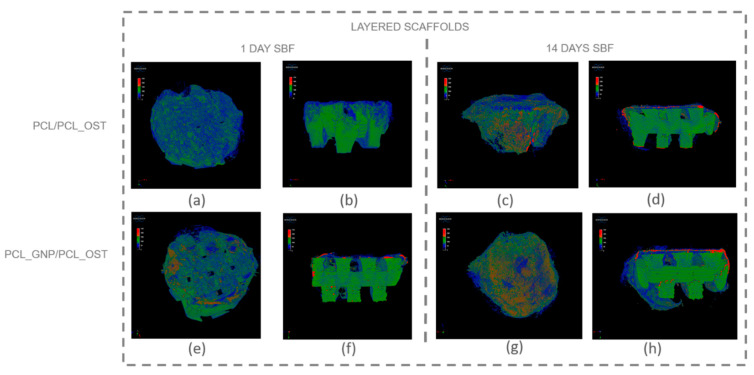
Micro-CT images of scaffolds after 1-day and 14-day SBF incubation: (**a**–**d**) PCL/PCL_OST scaffold; (**e**–**h**) PCL_GNP/PCL_OST scaffold. Images (**a**,**c**,**e**,**g**)—scaffolds top view, (**b**,**d**,**f**,**h**)—cross-sections. Bottom layer of 3D-printed scaffold marked in green, electrospun membrane marked in blue, and modifying particles, primarily distributed on the membrane, highlighted in red.

Micro-computed tomography (micro-CT) enables non-invasive, high-resolution, 3D visualization of the material internal structure. In this study, the micro-CT analysis was employed to examine the scaffold as a whole and also its individual layers—the bottom layer made of graphene-modified polycaprolactone (PCL) produced by 3D printing, and the top layer made of OST-modified PCL created via electrospinning. Figure 5 presents the μ-CT images of the scaffolds after 1 and 14 days of SBF incubation.

The internal structure of the scaffolds was clearly visualized. The layered scaffold comprised an electrospun membrane and an FDM-printed scaffold. The membrane constituted the upper layer of the scaffold, with portions extending around its sides. In the PCL_GNP/PCL_OST sample, apatite deposits were evident after just 1-day incubation. Apatite, predominantly forming on the electrospun membrane, was highlighted in red, the FDM-printed substrate was marked in green, and the applied membrane—in blue. After 14 days of incubation, the apatite formation was evident on both types of samples.

**Figure 6:**
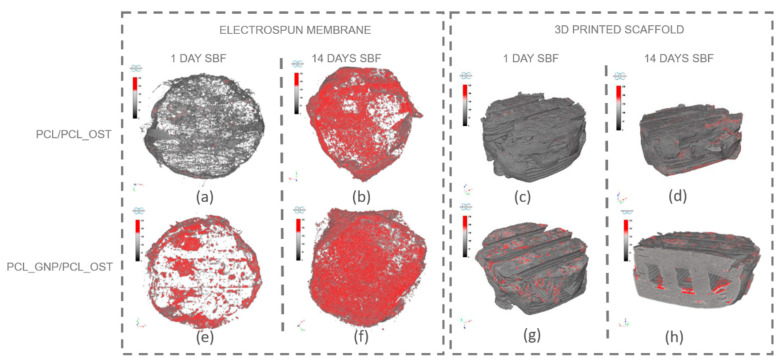
Separate micro-CT images of electrospun membranes and bottom layers of scaffolds for PCL/PCL_OST and PCL_GNP/PCL_OST samples after 1 and 14 days of incubation in SBF solution. Electrospun membranes of PCL/PCL_OST scaffold after 1 day (**a**) and 14 days (**b**); bottom layer of PCL/PCL_OST scaffold after 1 day (**c**) and 14 days (**d**). Electrospun membranes of PCL_GNP/PCL_OST scaffold after 1 day (**e**) and 14 days (**f**); bottom layer of PCL_GNP/PCL_OST scaffold after 1 day (**g**) and 14 days (**h**). Apatite formation on sample surfaces marked in red.

For a more detailed analysis, the membrane and the lower part of the scaffold were segmented to enable virtual reconstruction and identification of structural components on the electrospun membrane. The results of this analysis are presented in Figure 6 and Table 1.

The results presented in Figure 6 show that apatite deposits observed after 1 day of incubation appeared both on the membrane surface in the graphene-modified sample (Figure 6e) and on the surface of the 3D-printed scaffold (Figure 6g). After 14 days, apatite extensively covered the surface of both membranes (Figure 6b,f); it was also present on the 3D-printed scaffold (Figure 6d,h), and penetrated into the lattice structure of the graphene-modified implant (Figure 6h). The parameters evaluated for the electrospun membranes (upper layer of the scaffold) using micro-CT analysis are presented in Table 1.

The measured parameters included the object volume, surface area, structural thickness, and porosity of the electrospun membrane, as well as the number, volume, structural dimensions, and proportion of apatite inclusions within the membrane. The object volume of the membranes increased over time in SBF, indicating material swelling. Initially, the graphene-modified membranes had a smaller surface area (43.67 mm^2^) compared to the non-modified samples (86.07 mm^2^). Such results may stem from graphene altering the local electric field during electrospinning, affecting fiber deposition and potentially reducing the measured surface area. Conversely, the graphene-modified samples exhibited greater structural thickness after 1 day (74.76 µm). However, the thickness value stabilized or slightly decreased over time (65.74 µm), due to changes in swelling behavior and material interactions. Furthermore, the porosity decrease over time, especially evident in the graphene-modified samples (from 50.71 to 45.65%), may be attributed to the progressive growth of the apatite layer.

The PCL_GNP/PCL_OST samples exhibited a lower number of apatite objects (1181) in the membrane after 1-day SBF incubation, as compared to the PCL/PCL_OST samples (3610). This indicates that after 1-day incubation the GP samples already established numerous nucleation points and started forming larger apatite crystals. In contrast, the non-graphene samples were only beginning to develop nucleation points. This implies that graphene may accelerate the nucleation process and the subsequent growth of apatite crystals. Over time, the number of apatite objects decreased in both types of samples (1270 for PCL/PCL_OST and 839 for PCL_GNP/PCL_OST), indicating consolidation or growth of the existing apatite crystals. The volume of apatite objects was substantially larger in the PCL_GNP/PCL_OST samples, particularly after 14 days (0.91 mm^3^) than in PCL/PCL_OST (0.68 mm^3^). This proves the apatite growth over time in the presence of graphene. Additionally, the apatite structures were thicker in the graphene-modified samples after 1 day (77.02 µm), then the thickness either decreased slightly or stabilized after 14 days (53.59 µm). This suggests the initial rapid apatite deposition or growth that stabilized over time. The apatite volume fraction was much higher in the GNP-modified samples, especially after 1 day (59.76%), indicating that graphene significantly promoted the apatite deposition. Over time, this percentage stabilized (51.73), revealing a mature or fully developed apatite structure.

**Figure 7:**
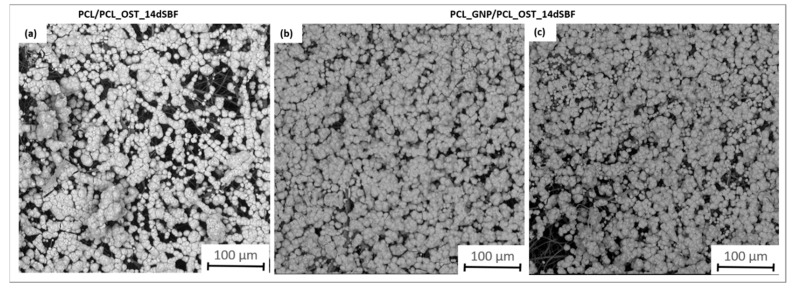
SEM images of samples after 14 days of immersion in SBF solution: (**a**) PCL/PCL_OST layered scaffold; (**b**,**c**) PCL_GNP/PCL_OST layered scaffold.

The SEM images (Figure 7) provide a detailed view of the apatite layer formed on the scaffolds after 14 days of incubation in SBF.

They apatite layer on the GNP sample was notably more compact; it uniformly covered the entire surface, as compared to the layer observed on PCL/PCL_OST. This enhanced coverage suggests more effective mineralization, likely attributed to the graphene presence, which promotes better nucleation and growth of apatite crystals. The SEM images also showed the granular texture of the apatite, indicating a well-developed crystalline structure. These observations were consistent with the micro-CT findings, highlighting the improved performance of the graphene-enhanced scaffold in facilitating the apatite formation.

**Figure 8:**
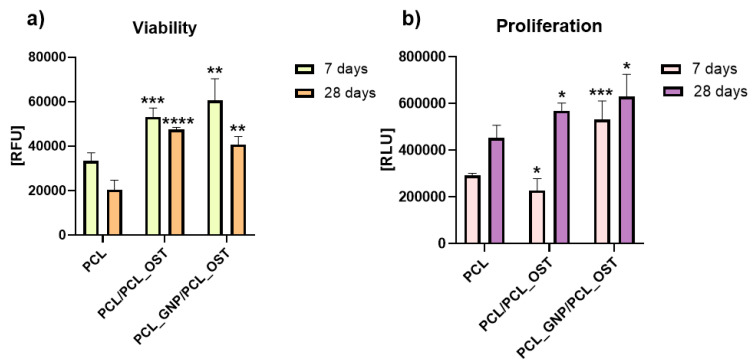
Cell viability (**a**) and proliferation (**b**) after 7 and 28 days of culture in direct contact with scaffold. Results expressed as the mean ± SD measured at least from 3 independent experiments. Significance determined with unpaired *t*-test. *p*-values of 0.05 or less considered statistically significant. * *p* values 0.05, ** *p* values 0.01, *** *p* values 0.001, **** *p* values 0.0001.

The cells were cultivated directly on the surface of the materials and the cell viability was measured after 7 and 28 days via PrestoBlue^TM^, Thermo Fisher Scientific Inc., USA according to the manufacturer protocol. The lowest viability was detected on the cells cultivated on PCL after 28 days (Figure 8).

Cells cultivated on PCL_GNP/PCL_OST revealed the highest viability after 7 days (Figure 8a). Unfortunately, after 28 days the cell viability on all the materials (PCL, PCL/PCL_OST and PCL_GNP/PCL_OST) was lower than after 7 days of culture.

The cell proliferation was evaluated using ToxiLight^TM^ bioassay kit (Lonza, Basel Stucki, Switzerland). After 7 days, in comparison to cells cultivated on PCL, the cell proliferation on the PCL/PCL_OST samples decreased (* *p* < 0.05) and on PCL_GNP/PCL_OST increased (*** *p* < 0.001) (Figure 8b). The tested materials revealed the cell proliferation increase after 28 days (* *p* < 0.05). The results proved that the addition of OST and GNP increased the proliferation after 28 days.

## 4. Discussion

In the literature, the integration of 3D printing and electrospinning technologies is highlighted for its advantages, particularly the potential for prototyping complex microstructures [46]. However, previous studies identified certain difficulties in selecting materials compatible with both methods and optimizing their processing parameters [47]. In our research, we were able to address these challenges. A novel bi-layered scaffold we designed effectively combined the benefits of both technologies. The macro- and microscopic observations of the 3D-printed scaffolds before and after electrospinning revealed their porous microstructure, the graphene incorporation and dispersion in the PCL_GNP samples, and the successful fabrication of a fibrous membrane from an OST-modified PCL solution on the surface of the 3D-printed samples. The mechanical tests showed that a small addition of graphene nanoplatelets (GNP) (0.5 wt%) to polycaprolactone (PCL) significantly increased the strain at break of the composite material without affecting its elastic modulus. It was observed that while the graphene addition did not change the PCL elastic modulus, it remarkably enhanced the material’s ability to deform, resulting in a much higher strain at break. This suggests that graphene can improve the PCL ductility, allowing the material to undergo more deformation before failure. Additionally, a slight increase in tensile strength indicates that graphene may also reinforce the polymer matrix.

These findings differ from other studies on graphene-based composite polymers which typically show an increase in tensile strength and elastic modulus, and reduced strain at break as the graphene content in the samples increases [48]. In most cases, the addition of graphene nanoplatelets (GNP) decreases the composite ductility by restricting the movement of polymer chains [49,50]. The incorporation of graphene into the polymer matrix enhances crosslinking between polymer chains, improving mechanical properties due to GNP exceptional mechanical characteristics.

However, the polymer chain movement is inhibited due to the GNP presence, which may lead to a decrease in strain at break in the nanocomposites. Therefore, numerous studies have shown that graphene/polymer nanocomposites exhibit significantly better mechanical properties than pure polymers [51]. When the loading of GNP exceeds 10 wt%, the mechanical properties decrease to those with lower filler content, although they remain significantly higher than those of the pure polymer. This might be attributed to the agglomeration of GNP in the polymer matrix, which can act as an impurity and degrade the mechanical properties.

In our case, the increased strain at break at low graphene addition may result from the GNP ability to distribute stress more evenly throughout the material, which prevents localized stress concentrations and allows the material to sustain higher strains before breaking. The effectiveness of these improvements depends on how well the graphene is dispersed within the polymer matrix and the specific conditions of the composite processing.

In this work, the calorimetric studies involved thermographic recognition of polycaprolactone and graphene used as a modifier. We examined how the form of PCL (granules, nanofibers) affected the supermolecular structure. We assessed the GNP influence on the crystalline phase content of PCL scaffolds, as well as its nucleation efficiency during the non-isothermal crystallization process during the scaffold printing. The calorimetric studies demonstrated that the form of polycaprolactone (PCL) significantly affected its supermolecular structure—nanofibers showed higher crystallinity than granules. The presence of graphene nanoplatelets (GNP) as a modifier did not substantially alter the crystalline phase content of PCL scaffolds, but it acted as an effective nucleating agent, shifting crystallization temperatures to higher values during the non-isothermal crystallization process [52,53].

The micro-CT studies revealed that the graphene-modified samples had fewer but larger apatite objects after 1 day incubation, proving faster nucleation and growth of apatite crystals in comparison to the non-modified samples. Over time, both sample types showed fewer apatite objects, suggesting consolidation or growth of existing crystals. However, the volume of apatite objects was larger in the graphene-modified samples, especially after 14 days, indicating the enhanced growth in the GNP presence.

The apatite thickness and volume fraction were initially higher in the graphene-modified samples but tended to stabilize over time, suggesting that graphene promoted faster and more extensive apatite formation. Graphene possesses a high surface area, which provides numerous nucleation sites that facilitate the initial formation of apatite crystals and accelerate the precipitation process. This extensive surface area allows more calcium and phosphate ions from SBF to interact with the scaffold, promoting faster apatite formation. Additionally, the polycarboxylate functional groups on GNP enhance the ion interaction by attracting and binding calcium and phosphate ions, which further accelerates mineralization. The hydrophilic nature GNP aids in maintaining a favorable environment for the ion interaction by improving the nanoparticles dispersion in the aqueous SBF solution. Graphene’s high electrical conductivity may also impact electrochemical processes on the material surface, enhancing the adsorption of ions and molecules from SBF onto the scaffold and facilitating rapid nucleation and growth of apatite crystals [54]. Furthermore, there may be a synergistic effect with Osteogenon^®^, where the physicochemical properties of graphene and the biological activity of Osteogenon^®^ collectively enhance apatite deposition. Moreover, the GNP surface chemistry can mimic certain characteristics of natural bone minerals, making the scaffold surface more conducive to the apatite growth by altering its surface energy to favor mineralization. The beneficial GNP effect on the apatite layer formation was also confirmed by the FTIR and SEM analyses after the SBF incubation.

Research conducted by other scientists has demonstrated that graphene exhibits good cytocompatibility both in vitro and in vivo. Graphene’s physical and chemical characteristics including morphology, structural arrangement, size, surface functionality, concentration, and aggregation state, significantly influence cellular behavior. The graphene nanoplates upregulate the phosphorylated level of Akt and inhibit the proteasomal degradation of b-catenin and promote the expression of osteogenic-related genes [55]. Consequently, graphene-based materials serve as valuable substrates for cell growth [56]. Pristine graphene has been shown to enhance the attachment, proliferation, and differentiation of various cell types [57,58].

Graphene exhibits its effectiveness by anchoring calcium ions, enhancing mineralization, and promoting cell differentiation. Additionally, its composites can increase cell migration and new tissue formation [56,59].

In our study, the combined use of GNP and OST components in PCL scaffolds created a favorable environment for chondrocytes, increasing the cell viability and proliferation. OST supplied biochemical signals crucial for tissue repair. The synergy between graphene nanoparticles (GNPs) and Osteogenon^®^ (OST) is hypothesized to enhance both antibacterial and regenerative performance through complementary mechanisms. GNPs possess inherent antibacterial activity, attributed to their sharp edges and ability to induce oxidative stress in bacterial membranes, while also supporting cell adhesion and proliferation. Simultaneously, OST provides essential osteoconductive and osteoinductive cues that stimulate mineralization and bone tissue formation. When combined, GNPs may help prevent bacterial colonization at the implant site, while OST promotes efficient tissue regeneration, making the composite scaffold highly suitable for use in bone repair and regenerative medicine.

The synergy between GNP and OST resulted in the improved cellular behavior observed in our study.

## 5. Conclusions

A novel bi-layer scaffold for osteochondral reconstruction was successfully developed using 3D printing and electrospinning, incorporating graphene for antibacterial properties and Osteogenon^®^ to support bone regeneration. Incorporation of 0.5% graphene nanoplatelets (GNP) into PCL significantly enhanced ductility, increasing the strain at break approximately threefold (to 78%) compared to pure PCL, while maintaining a similar elastic modulus (~136 MPa) in both PCL and PCL_GNP composites. GNP functioned as a nucleating agent, raising crystallization temperatures, and promoting mineralization, thereby highlighting its potential for tissue engineering.

Graphene’s high surface area accelerated the apatite formation by attracting calcium and phosphate ions, facilitating faster and more extensive mineralization. The modified scaffolds demonstrated significantly improved biological performance, with all the samples showing the increased chondrocyte viability and proliferation, especially in the presence of osteogenon modifications and graphene nanoparticles. Viability noticeably improved both at 7 and 28 days compared to pure PCL. The synergistic effects of GNP and OST created a bioactive environment that enhanced cell adhesion and proliferation. Additionally, the combined effect of graphene’s physicochemical properties and Osteogenon^®^ bioactivity contributed to superior apatite formation, evidenced by a 33.8% higher apatite volume in PCL_GNP/PCL_OST samples compared to PCL/PCL_OST after 14 days, reinforcing the scaffold’s potential in regenerative medicine. These findings indicate that the developed scaffold could serve as a promising biomaterial for osteochondral repair. Future studies should aim to refine the osteochondral interface model and finalize a patient-specific implant design. It is also necessary to investigate how GNP/OST modifications influence mesenchymal stem cell differentiation into chondrocytes and osteoblasts. Ultimately, in vivo studies will be essential to evaluate long-term biocompatibility, degradation behavior, and mechanical stability under physiological conditions.

## Figures and Tables

**Table 1:** 3D analysis parameters of upper scaffold layer produced via electrospinning (membrane) for samples after 1 and 14 days of incubation, along with apatite morphological analysis.

Electrospun Membranes (Scaffold Upper Layer)				
	1 Day SBF		14 Days SBF	
	PCL /PCL_OST	PCL_GNP /PCL_OST	PCL /PCL_OST	PCL_GNP /PCL_OST
Obj.V ^1^ [mm^3^]	0.72	0.71	1.36	1.76
Obj.S ^2^ [mm^2^]	86.07	43.67	84.08	93.60
St.Th ^3^ [µm]	35.62	74.76	62.28	65.74
Po(tot) ^4^ [%]	74.77	50.71	65.96	45.65
**Apatite in Membrane**				
Obj.N ^5^	3610	1181	1270	839
Obj.V ^1^ [mm^3^]	0.01	0.43	0.68	0.91
St.Th ^3^ [µm]	48.21	77.02	55.75	53.59
Obj.V/TV ^6^ [%]	1.41	59.76	50.24	51.73

^1^ Object volume, ^2^ object surface, ^3^ structure thickness, ^4^ membrane porosity, ^5^ number of objects, and ^6^ object volume/total volume.

## Data Availability

The original contributions presented in this study are included in the article. Further inquiries can be directed to the corresponding author.
